# A 3D Relative-Motion Context Constraint-Based MAP Solution for Multiple-Object Tracking Problems

**DOI:** 10.3390/s18072363

**Published:** 2018-07-20

**Authors:** Zhongli Wang, Litong Fan, Baigen Cai

**Affiliations:** 1 School of Electronic Information and Engineering, Beijing Jiaotong University, Beijing 100044, China; 15120240@bjtu.edu.cn (L.F.); bgcai@bjtu.edu.cn (B.C.); 2 Beijing Engineering Research Center of EMC and GNSS Technology for Rail Transportation, Beijing 100044, China; 3 School of Computer Information Management, Inner Mongolia University of Finance and Economics, Hohot 010010, China

**Keywords:** tracking by detection, 3D relative-motion model, sequential Bayesian framework, multi-object tracking

## Abstract

Multi-object tracking (MOT), especially by using a moving monocular camera, is a very challenging task in the field of visual object tracking. To tackle this problem, the traditional tracking-by-detection-based method is heavily dependent on detection results. Occlusion and mis-detections will often lead to tracklets or drifting. In this paper, the tasks of MOT and camera motion estimation are formulated as finding a maximum a posteriori (MAP) solution of joint probability and synchronously solved in a unified framework. To improve performance, we incorporate the three-dimensional (3D) relative-motion model into a sequential Bayesian framework to track multiple objects and the camera’s ego-motion estimation. A 3D relative-motion model that describes spatial relations among objects is exploited for predicting object states robustly and recovering objects when occlusion and mis-detections occur. Reversible jump Markov chain Monte Carlo (RJMCMC) particle filtering is applied to solve the posteriori estimation problem. Both quantitative and qualitative experiments with benchmark datasets and video collected on campus were conducted, which confirms that the proposed method is outperformed in many evaluation metrics.

## 1. Introduction

Multiple object tracking on a moving platform is an active research topic in computer vision, with applications such as automotive driver assistance systems, robot navigation and traffic safety. To date, much progress has been made in this field. Tracking-by-detection is one of the more extensively investigated methods. It formulates the problem as associating detection results with each other across video sequences to get the trajectories and identities of the targets. Traditional tracking-by-detection methods are heavily dependent on detection results, because there are many factors, such as inaccurate detections, similarity between objects, frequent occlusion, etc., which lead to tracklets or tracking drift, making multi-object tracking (MOT) still a very challenging task.

To solve the problem, many approaches have been presented during past decades. Some trackers explore different tracking algorithms to connect the detected targets’ positions and form trajectories. The tracking algorithms generally use associations or build an affinity model to connect the detected target, even predicting the positions of targets in the next frame. All existing tracking-by-detection approaches can be loosely categorized into batch mode [1,2,3,4,5,6,7] or online mode [8,9,10,11,12,13,14,15,16,17,18,19,20,21,22], depending on the frames used by the tracker.

Batch mode-based methods utilize the detection of entire sequences for global association to establish a robust target trajectory, so as to compensate against occlusion and false detections. The global association is realized by a sliding window or hierarchical association, and often formulated as a graph optimization problem. It models the tracking problem as a network flow and solves it either with k-shortest paths in dynamic programing with non-maximum suppression (DP NMS) [1], as a Generalized Maximum Multi-Clique problem which is solved with Binary Integer Programing in reference [2], in a conditional random field (as in SegTrack) [3], with Long-term time-sensitive costs for CRF(LTTSC-CRF) [4], or with discrete-continuous energy minimization [5], to name a few. These algorithms can correct previous data association errors and recover observations from an occluded state by using a future frame for association. Deep learning has also been applied to tracking for robust link detections; however, its application to the problem at hand remains rather sparse. One example is the jump Markov chain (JMC) method in [6], which uses deep matching to improve affinity measurement, Siamese-convolutional neural network (CNN) uses a CNN in a Siamese configuration to estimate the likelihood that two pedestrian detections belong to the same tracked entity [7]. However, these kinds of methods usually need detections for the entire sequence as input, only then generating globally optimized tracks by iterative associations. It is difficult to apply batch methods to real-time applications due to the expensive computation and special input required.

On the other hand, online mode-based methods [8,9,10,11,12,13,14,15,16,17,18,19,20,21,22] take real-time applications into consideration. Such methods use information up to the current frame, and link the detections frame by frame to build trajectories. This method solves the association problem by Bayesian filter or local data association, such as the Markov chain Monte Carlo (MCMC) filter [8,9], Kalman filter [10,11], or probability hypothesis density (PHD) filter [12,13]. The tracking problem is formulated as a Bayesian framework given past and current measurements to find the maximum-a-posteriori (MAP) solution. However, online methods are prone to producing fragmented tracklets and drifting with occlusion and detection errors, as it is more difficult to cope with inaccurate detections (such as false positives and false negatives) compared with the batch methods [14]. Many online methods have been proposed to solve the track fragments and tracking drift problems due to inaccurate detections. Some attention shifted towards building robust pairwise similarity costs, mostly based on strong appearance cues. For example, the discriminative deep appearance learning in reference [14], integral channel feature appearance models in online integral channel features (oICF) [15], and temporal dynamic appearance models in Temporal dynamic appearance modeling (TDAM) [16] are proposed to improve detection affinity, while utilizing markov decision process neural network (MDPNN16) [17], which leverages Recurrent Neural Networks, in order to encode appearance, motion, and interactions. Comparatively, single object tracking (SOT) is another field of active research [18], where correlation filters (CFs) may be the mainstream in this field. Many enhanced methods, such as output constraint transfer for kernelized correlation filter (OCT_KCF) [19] and latent constrained correlation filter (LCCF) [20] are proposed to further improve the performance on model drifting or other aspects. In reference [21], hierarchical convolutional features are used for the adaptive correlation filters to address the appearance variation, another correlation filter is designed for scale estimation and target re-detection in case of tracking failure, the latter is necessary for long-term tracking tasks. Though CF-based methods show impressive performance, but most of the designed trackers are limited to single object tracking currently. How to extend CF to MOT is still an open issue. Further attention has been given to motion models of the tracked objects, which can be exploited to predict the target position. Relative motion network (RMN) [22], for instance, constructs a relative motion network that accounts for the relative movement among objects to predict the objects’ positions when detection fails and to handle unexpected camera motion for robust tracking.

In the scenarios we address in this paper, the monocular camera is moving and there are moving targets in the scene. This brings great challenges to the problem. Reconstructing the scene structure while estimating the camera’s motion simultaneously is the well-studied structure-from-motion (SFM) problem, wherein the scene is stationary in the computer vision community. But when the camera is moving, the motions of tracked objects will couple with the camera’s motion. To tackle this problem, we need to estimate the camera’s motion along with other stationary features, so we need to separate the extracted stationary features from the dynamic ones, which allows us to estimate the camera’s motion robustly and compensate for target motion.

Additionally, we focus on the online method to address the fragmented trajectories and drifting caused by inaccurate detection. The relative-motion context of targets in 3D space is proposed for target association, which describes the 3D movement relationships among the tracked targets. Relatively speaking, the relative motion in two-dimensional (2D) imaging loses its depth information and can’t describe the relative positions of targets as accurately as in 3D space. Furthermore, in the case of camera motion, we incorporate a relative-motion model into a sequential Bayesian framework to track multiple objects and estimate the ego-motion. Because the number of targets changes dynamically, we use a sample method with reversible jump Markov chain Monte Carlo (RJMCMC), which unifies all modules under the Bayesian framework.

**Figure 1:**
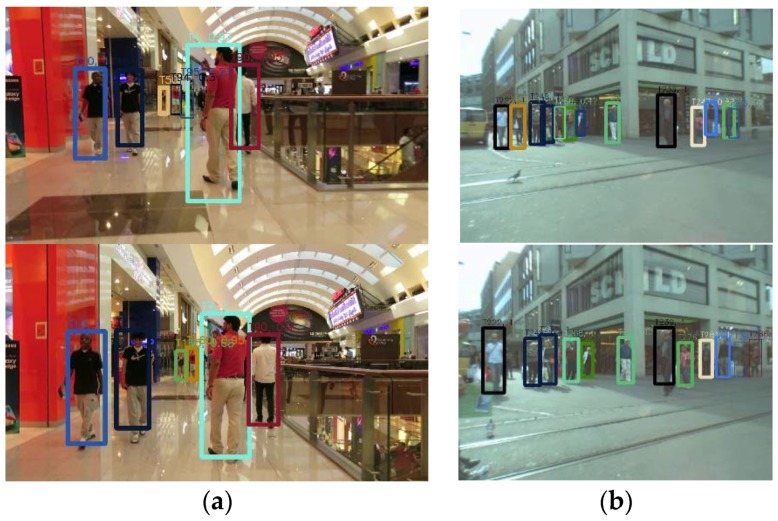
Some tracking results with the proposed method. (**a**) Tracking results of the 23rd frame and 49th frame of MOT16-11. (**b**) Tracking results of 78th frame and 94th frame of ETH-Linthescher.

The main contributions of our work include: (1) A 3D relative-motion model is proposed to improve the track fragments due to occlusion or unreliable detections, which is more intuitive and reasonable for motion prediction; (2) Based on the 3D relative-motion model, an unified Bayesian framework is presented, which combines the MOT problem with camera motion estimation, and formulates it as finding a MAP solution of joint probability; (3) Validation of the proposed method with standard dataset and real data collected in the campus. Figure 1 shows some outperforming results of the proposed method. In this figure, the tracked objects are marked with different colors. It shows that the proposed method can track targets even in a crowded scene.

The paper is organized as follows. The related works are reviewed in Section 2. Section 3 describes the unified Bayesian model and key parts of the proposed method. The proposal distributions for the models are introduced in Section 4. Experimental results are presented in Section 5 and followed by conclusions in Section 6.

## 2. Related Works

Because we focused on online multiple object tracking (MOT) with a moving camera, target motion models are exploited to enhance the proposed method’s robustness. The literature on multiple object tracking with a moving camera and the existing motion models are thus briefly summarized.

### 2.1. MOT with a Moving Camera

Several representative MOT algorithms that focus on online methods using a moving monocular camera have recently been proposed, which address the challenges of targets tracking from a moving platform. Some of them aim at solving unreliable detections by modeling the detection confidence [23,24,25]. Breitenstein [23] combines online-trained, instance-specific classifiers as a graded observation model and particle filtering for robust multi-person tracking suitable for online applications. Within a probability hypothesis density particle filter framework, early association multiple target tracking (EAMTT) [24] exploits both high- and low-confidence target detections, wherein the high confidence detections and low confidence detections are used for different purposes. Some methods solve the issue of tracking from a mobile platform by aiming at the introduction of a robust feature. For example, interaction feature strings that encode the physical interactions between targets are proposed in reference [25], and a TDAM method [16] is presented, which uses the temporal dynamic appearance model to encode the spatial structure and temporal dynamics of human appearance. Reference [14] combines two of the confidence-based associations for handling fragments and discriminative deep appearance learning for handling appearance variations.

There is also a kind of method that exploits relative motion to deal with unexpected camera motion. A relative-motion network (RMN) is proposed in [22], which encodes the motion context from multiple moving targets for the scenarios, during which the camera experiences small fluctuations. In reference [26], based on RMN, an improved method, Structural Constraint Event Aggregation, is proposed to handle abrupt camera motions and fluctuations. All these methods depend on improving association accuracy to compensate for the influence of camera motion. In reference [27], a framework which estimates camera motion while tracking is presented. Because the camera’s ego-motion is estimated during the tracking process, it is suitable for tracking multiple objects with a moving camera.

An expectation association (EA) algorithm is proposed in [28,29], which formulates multi-object tracking and classification as a MAP problem and proposes a variational approximation method for solving it. This work represents the motion patterns of the targets with a simple linear dynamical system (LDS), and a training process is necessary for the object class assignment; it limits the applicability of the method.

### 2.2. Motion Models in Tracking

Previous methods utilize both appearance and motion models for calculating affinity function. Due to the limitations of the appearance models used for tracking targets with similar appearances and target-to-target interactions, adding motion clues to predict the potential position of targets in future frames can reduce the search space, and hence, increase the accuracy of the appearance model. Generally, motion models used in the literature can be divided into linear and non-linear motion models.

Based on the assumption that a target’s motion within a short period of time is linear, the linear motion model uses first-order linear regression and is widely used in multi-target tracking [14,23,30,31,32]. However, there are many cases the linear motion model cannot handle, such as long-term occlusions, camera movement or fluctuation, or crowded scenes. It is obvious that non-linear motion models can produce a more accurate prediction [33,34] at the cost of a higher computing load. Usually, as all tracked targets move together when the camera moves, relative positions among the targets are less affected by the camera’s view angles. They may be more stable than linear motion models in position estimation under moving camera conditions [22,26,35]. The social force model (SFM) has been used for abnormal crowd behavior detection, collision avoidance, or group attraction [25].

Though the existing works show that by making use of motion context, the accuracy and robustness of tracking results can be improved, the motion models are still only exploited in a 2D image plane. Our experiments show that because of the loss in depth information in 2D imagery, the tracked target ID may be incorrect or missing for a same object in a crowded or occlusion scene. In this work, we assume the camera is moving along a horizontal plane. A 3D motion network model is exploited to reduce tracking drifts during occlusion or drastic appearance changes.

## 3. The Proposed Method

**Figure 2:**
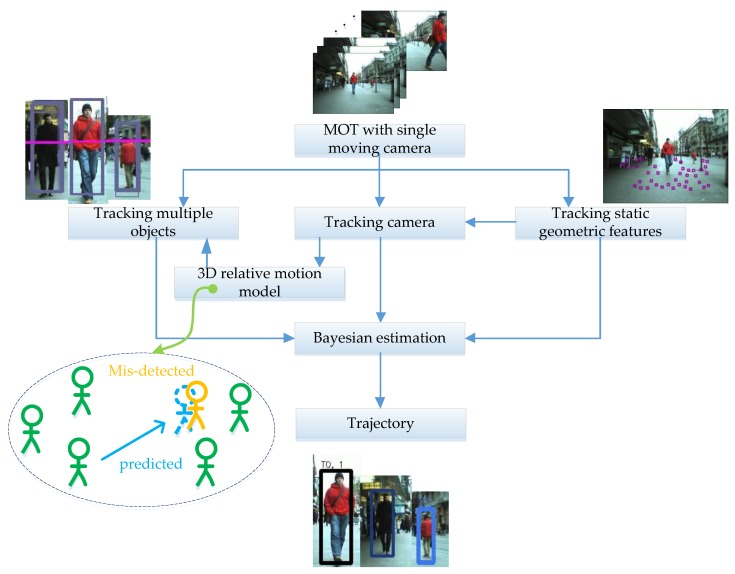
The scheme of the proposed method.

To address multi-target tracking issues with a moving camera, a Bayes posterior estimation-based MOT method was proposed under the framework of tracking by detection. The data processing flow of the proposed method is shown in Figure 2. The Bayes posterior estimation-based method uses observation cues to generate a proposal, then uses the observation likelihood and relative-motion models to estimate a proposal to generate the trajectory. The observation cues are provided by different detectors, and then the observation likelihood between observation cues and the target’s hypothesized position is computed. The target hypotheses are predicted by a relative-motion model in 3D space. A 3D relative-motion model describes the relative movement between targets three-dimensionally, which is more accurate and intuitive than the 2D motion network in a single image plane. We exploited the camera model to establish the relationship between the targets in 3D space and in the 2D image plane to compute a target’s motion model observational likelihood. Because the camera is moving, we estimated the camera’s motion by estimating stationary geometric features, then projecting targets’ hypothesized positions into the image plane at each step.

This method fuses multiple detection clues to adapt to changes in target appearance and exploration of a complex environment, and utilizes the relative-motion model in 3D space to resolve track fragments and tracking drift due to mis-detection and occlusion. Our method could also adapt to the complex motion of a target and reduce the dependence of the tracking algorithm on the detection results. RJMCMC particle filtering was exploited to solve the posterior estimation problem. It can add or remove targets randomly via the reversible jump move operation, and adapts to random changes in the number of targets.

### 3.1. The Unified Bayesian Framework

The tracking task and camera motion estimation are unified with a Bayesian framework and formulated as finding the maximum a posteriori (MAP) solution of the joint probability. There are three types of parameters that need to be estimated: the camera parameter $\Theta_{t}$, a set of targets’ states $O_{t}$, and the geometric features’ states $G_{t}$. All these parameters are expressed as random variables and the relationships among them are expressed by the joint posterior probability. The approximate posterior distribution can be obtained with the RJMCMC method. The basic process is discussed as follows.

Given an image sequence $I_{0,\ldots t}$ at time (0, 1, 2, ... *t*), and the configurations of all the parameters at time *t* are written by $\chi_{t}=\left\{\Theta_{t},O_{t},G_{t}\right\}$, the goal is to find the best match configuration. The MAP problem $P(\chi_{t}|I_{0,\ldots t})$ is factored into several parts as follows:

(1) $$P(\chi_{t}|I_{0,\ldots ,t})\propto \underset{A}{P(I_{t}|\chi_{t})}\int \underset{B}{P(\chi_{t}|\chi_{t-1})}\underset{C}{P(\chi_{t-1}|I_{0,\ldots ,t-1})}d\chi_{t-1}$$

The first term indicated by *A* in Equation (1) defines the observation likelihood, which models the similarity between configuration $\chi_{t}$ and the sensory input $I_{t}$ at time *t*. This part measures how well the observations support the hypothesis for the current configuration. The second term indicated by *B* in Equation (1) is related to the motion model, which predicts the current state $\chi_{t}$ from the previous state $\chi_{t-1}$ under the Markov assumption conditions, and is composed of motion model, shape change, and interaction between objects. The third term indicated by *C* is the prior, which is actually the posterior probability from the previous time step.

### 3.2. Camera Model and Camera Motion Estimation

(1) Camera Model

We adopted a camera model similar to reference [36], and set up the relationship of targets in 3D space and a 2D image, given the image’s horizon location and the camera’s height beforehand. The parameters of the camera Θ include: focal length *f*, horizon line $v_{h}$, image center $u_{c}$, yaw angle φ, camera height $h_{\Theta }$, velocity μ, and 3D location $\left(x_{\Theta },z_{\Theta }\right)$. The projection function $f_{\Theta_{t}}$, which expressed the relationship between camera parameters $\Theta_{t}$ and object location *O* was defined as:

(2) $$X=\left[\begin{array}{l} x\\ y\\ h \end{array}\right]=f(O)=\left[\begin{array}{l} \frac{fx_{z}}{z_{z}}+u\\ \frac{fH}{z_{z}}+v\\ \frac{fh_{z}}{z_{z}} \end{array}\right],\;O_{0}=\left[\begin{array}{cc} R(\phi ) & 0\\ 0 & 1 \end{array}\right]O+\left[\begin{array}{c} x_{\Theta }\\ z_{\Theta }\\ 0 \end{array}\right]$$

where $O_{0}$ is the position of target in the present camera coordinates, and X=(x,y,h) refers to the location and height of the corresponding bounding box in the image plane, respectively. The projection function is also suitable for geometric features, such as the projected location for targets’ feet.

(2) Camera Motion Estimation

The camera motion was estimated first in the proposed framework, which is used for obtaining the current camera state, and removing the influence of camera motion to calculate the target motion between two frames. This estimation is composed of two parts, the camera motion priori and geometric feature based motion estimation.

Camera motion priori. A dynamic model with a constant velocity was employed to model camera motion, which was based on a reasonable assumption that the motion of a camera is smooth over a short time. In terms of robustness, an error item is included to account for uncertainty in the simplified camera model. This constant perturbation model was applied to the parameters, such as camera height, horizon, velocity, and yaw angle, etc.: $\varphi_{t+1}=\varphi_{t}+\varepsilon$. The location updating process can be written as:

(3) $$x_{t+1}=x_{t}+v_{t}\cos(\varphi_{t})+\varepsilon ,z_{t+1}=z_{t}+v_{t}\sin(\varphi_{t})+\varepsilon$$

The intrinsic camera parameters, such as focal length, skewness, optical center, etc., are assumed to be known and provided by default.

Geometric feature-based motion estimation. Feature tracking was the fundamental of motion estimation in this work. To balance the efficiency and the performance, Speed-Up Robust Features (SURF) detector [37] was used for feature descriptor, and the Kanade-Lucas-Tomasi Feature Tracker (KLT) tracker [38] was employed for the feature tracking of adjacent frames. Because the camera is moving, the extracted geometric features consist of static features from the background and dynamic ones from the moving targets, but only the static features will be used for the camera motion estimation. Therefore, removing the dynamic features from all extracted features is very important for the camera motion estimation. Based on the results of KLT tracker, some constraints were used for selecting the static features first.

Generally, we assumed that the valid static features are all below the horizon line [39] because the platform with camera was moving on a flat ground, and not on the target. To avoid the concentration of the selected features, the distances among the features should be larger than a threshold. Usually, we can select out many points for the next step, but only about 40 feature points are used for the efficiency. Among these left points, there may exist a few of mismatched feature points which will affect the motion estimation. Then the validity of the left features will be further verified by the motion prior and observation likelihood as follows.

The motion priori of the geometric feature. The motion priori encodes smooth transition between features of adjacent frames, and describes whether geometric features’ positions are consistent with their previous locations. Correspondently, it also describes the possibility of static or moving states. Let $P_{val}^{}$ be the validity prior to describe the features as static or not static, and $P_{cons}^{}$ be the consistency prior. Then, we can get:

(4) $$P(G_{t}|G_{t-1})=P_{val}(G_{t}|G_{t-1})P_{cons}(G_{t}|G_{t-1})$$

Two binomial probabilities are included, consisting of the probability of staying in the scene $p_{s}^{g}$ and the probability of entering the scene $p_{e}^{g}$, to model the prior validity respectively. Basically, these variables can represent the probability that a valid geometric feature will likely remain valid in the next frame. Then the validity prior can be written as:

(5) $$P_{val}(G_{t}|G_{t-1})=\prod_{j}P_{val}(G_{t}^{j}|G_{t-1}^{j})$$

(6) $$P_{val}(G_{t}^{j}|G_{t-1}^{j})=\left\{ \begin{array}{cc} p_{s}^{g} & \mathrm{if}\;j\;\mathrm{is}\;\mathrm{valid}\;\mathrm{at}\;t-1\;\mathrm{and}\;t\\ 1-p_{s}^{g} & \mathrm{if}\;j\;\mathrm{is}\;\mathrm{valid}\;\mathrm{at}\;t-1\;\mathrm{but}\;\mathrm{not}\;t\\ p_{e}^{g} & \mathrm{if}\;j\;\mathrm{is}\;\mathrm{valid}\;\mathrm{at}\;t\;\mathrm{but}\;\mathrm{not}\;t-1\\ 1-p_{e}^{g} & \mathrm{if}\;j\;\mathrm{is}\;\mathrm{not}\;\mathrm{valid}\;\mathrm{at}\;t\;\mathrm{but}\;\mathrm{not}\;t-1 \end{array}\right.$$

As the features are defined as static 3D world points, an indicator function is exploited to model a single feature’s priori of consistency $P_{cons}(G_{t}|G_{t-1})$:

(7) $$P_{cons}(G_{t}|G_{t-1})=\prod_{j}I(G_{t}^{j}=G_{t-1}^{j})$$

Geometric feature observation likelihood. The likelihood calculation process is completed by two steps: projecting the feature in 3D space to the image plane, and then detecting the feature using a detector. The point of interest in the image plane corresponding to a geometric feature $G_{t}^{i}$ is represented as $\tau_{t}^{i}$, the likelihood is modeled as a normal Gauss distribution centered on the projection of feature $f_{\Theta }(G_{t}^{i})$, and we introduce a uniform background model for invalid features, such as occluded or non-stationary features. Equation (9) defines the function for measuring geometric feature likelihood.

(8) $$P(I_{t}|G_{t}^{i},\Theta_{t})=P(I_{t}|f_{\Theta_{t}}(G_{t}^{i}))$$

(9) $$P(I_{t}|f_{\Theta_{t}}(G_{t}^{i}))=\left\{ \begin{array}{cc} N(\tau_{t}^{i};f_{\Theta_{t}}(G_{t}^{i}),\sum_{G}) & G_{t}^{i}\;\mathrm{is}\;\mathrm{valid}\\ K_{B} & G_{t}^{i}\;\mathrm{is}\;\mathrm{invalid} \end{array}\right.$$

By the combination of Gauss distribution and uniform background model, which can exclude the outliers with the maximum possibility, the robustness of the estimated camera motion can be improved.

Through the validity prior and consistency prior, the invalid and inconsistency features were removed. Only the valid static feature points were considered for the camera motion estimation.

### 3.3. 3D Relative Motion Model Based MOT

After the camera state and motion have been estimated, the estimated camera motion can be applied to the motion compensation for the MOT with the moving camera. With the unified Bayesian framework expressed in Equation (1), the observation likelihood and motion prior for the MOT problem then needs to be calculated. The proposed 3D relative motion model was applied for the motion prior estimation, which can make the motion model more robust and flexible. The observation likelihood model measured target hypotheses generated by motion priori that matched the input data (detection) best.

(1) The observation likelihood model

The observation likelihood model provides a measurement for evaluating which configuration *X_t_* matches the input data *I_t_* best. Configuration *X_t_* is provided by a set of hypotheses generated by prior motion, representing the true state of targets and geometric features in 3D coordinates. The input data *I_t_* is given by the detectors in the way of bounding box. Hence, the evaluation should transform the two variables into one coordinate.

**Figure 3:**
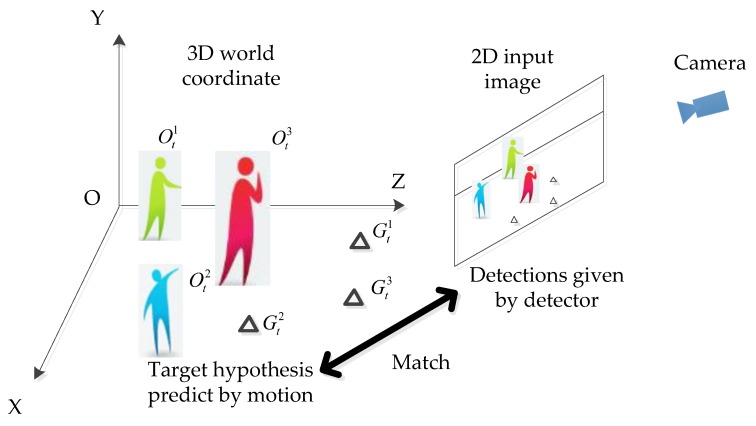
Observation likelihood model.

As shown in Figure 3, given a hypothesis for the target ${\hat{O}}_{t}$, we can evaluate the following two steps: (1) using the camera model to project each hypothesized target into the image, the projecting function is expressed as $f_{\Theta_{t}}$, and then (2) evaluating the observation likelihood given the detection cues. These two steps can be described in Equations (10) and (11). Here the likelihood between hypothesis targets and detection cues was calculated by the Hungarian algorithm as in reference [40]. The detection cues were provided by the deformable part model (DPM) detector [41] and the mean-Shift tracker [42].

(10) $$P(I_{t}|O_{t}^{i},\Theta_{t})=P(I_{t}|f_{\Theta_{t}}(O_{t}^{i}))$$

(11) $$P(I_{t}|\chi_{t})=\prod_{i}P(I_{t}|O_{t}^{i},\Theta_{t})\prod_{j}P(I_{t}|G_{t}^{j},\Theta_{t})$$

After re-projecting the target’s hypothesized position back to the image, we obtained the observation likelihood measurements both for the validity of the target and the accuracy of the location. The accuracy of targets is modeled via the observation likelihood $P(I_{t}|f_{\Theta_{t}}(O_{t}^{i}))$. Two detectors are employed as observation cues for various appearances. For simplicity, instead of using the likelihood $P_{j}$, log likelihood $l_{j}$ is adopted for each detector *j* with weight $w_{j}$ as:

(12) $$P(I_{t}|f_{\Theta_{t}}(O_{t}^{i}))\propto \exp(\sum_{j}w_{j}l_{j}(I_{t}|f_{\Theta_{t}}(O_{t}^{i})))$$

It is difficult to describe the possibility of a target actually existing or not. Here, the ratio of the likelihoods $P(I_{t}|f_{\Theta }(O_{t}^{i}))/P(I_{t}|f_{\Theta }(\phi ))$ is adopted for the validity measurement. It can allow the target states variable $O_{t}^{i}$ to have different dimensions. However, as the likelihood of an empty set is ambiguous, the ratio is modeled with a soft max *g*(◦) of the hypothesis likelihood, because it is robust against sporadic noise:

(13) $$\frac{P(I_{t}|f_{\Theta }(O_{t}^{i}))}{P(I_{t}|f_{\Theta }(\phi ))}\propto \exp\left(\sum_{j}g(w_{j}l_{j}(I_{t}|f_{\Theta }(O_{t}^{i})))\right)$$

(2) 3D Relative Motion Model and Motion Priori

The term for motion priori $P(\chi_{t}|\chi_{t-1})$ in Equation (1) is the evolution of the configuration between time steps which is consist of three parts: (1) camera’s prior motion; (2) target’s prior motion; or (3) geometric feature’s prior motion. It can be expressed as follows:

(14) $$P(\chi_{t}|\chi_{t-1})=P(O_{t}|O_{t-1})P(G_{t}|G_{t-1})P(\Theta_{t}|\Theta_{t-1})$$

The camera motion prior and geometric feature motion prior are discussed in the section of camera motion estimation. Here we focused on the proposed 3D relative motion model and the target motion prior.

To describe the relative-motion model among objects in 3D space, both the spatial and velocity information are considered. Let $O_{t}^{i}={\left[x_{t}^{i},y_{t}^{i},z_{t}^{i},{\dot{x}}_{t}^{i},{\dot{y}}_{t}^{i},{\dot{z}}_{t}^{i}\right]}^{T}$ is the state of object *i* at time *t*, where ($x_{t}^{i}$, $y_{t}^{i}$, $z_{t}^{i}$) and (${\dot{x}}_{t}^{i}$, ${\dot{y}}_{t}^{i}$, $z_{t}^{i}$) denote the 3D position and velocity respectively. Then the relative-motion model between two objects *i* and *j* is defined based on the position and velocity difference as:

(15) $$\begin{array}{l} r_{t}^{\langle i,j\rangle }≜r(o_{t}^{i},o_{t}^{j})\\ \;=\;[u_{t}^{\langle i,j\rangle },v_{t}^{\langle i,j\rangle },w_{t}^{\langle i,j\rangle },{\dot{u}}_{t}^{\langle i,j\rangle },{\dot{v}}_{t}^{\langle i,j\rangle },{\dot{w}}_{t}^{\langle i,j\rangle }{]}^{T}\\ \;=\;[x_{t}^{i}-x_{t}^{j},y_{t}^{i}-y_{t}^{j},z_{t}^{i}-z_{t}^{j},{\dot{x}}_{t}^{i}-{\dot{x}}_{t}^{j},{\dot{y}}_{t}^{i}-{\dot{y}}_{t}^{j},{\dot{z}}_{t}^{i}-{\dot{z}}_{t}^{j}{]}^{T} \end{array}$$

Then, the relative-motion model for N objects $R_{t}$ is then defined as:

(16) $$R_{t}=\cup_{i=1}^{N}R_{t}^{i},\;\mathrm{and}\;R_{t}^{i}=\{\langle i,i\rangle \}\cup \{\langle i,j\rangle |d_{t}^{j}=1,1\leq j\leq N\}.$$

**Figure 4:**
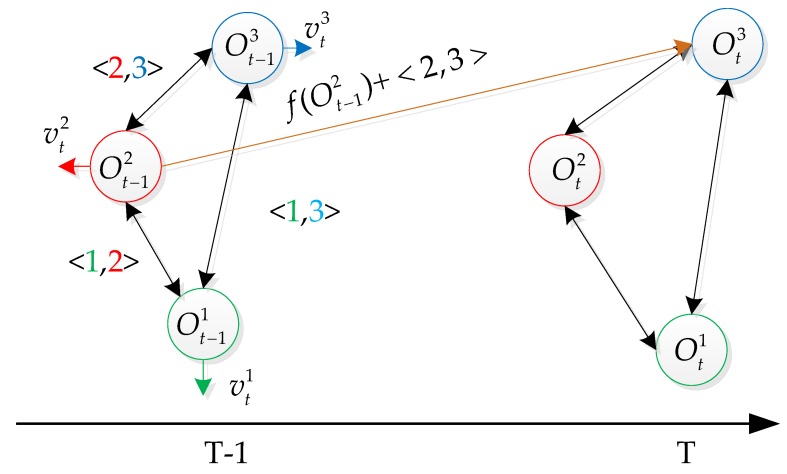
Predicting the target state with the relative-motion model.

The 3D relative-motion model actually represents a set of linked edges between objects. As illustrated in Figure 4, the states of different targets are represented as circles of different colors, the relative motion model 〈i,j〉 represents two different targets (*i*, *j*), and is denoted as black lines. While the relative motion model 〈i,i〉 encodes the self-motion model of target *i*, which is always included in case that there exists only one target. Usually, there are more than one relative motion for a target *i*. We chose the closest target *j* to target *i* that had detection assigned for predicting target $o_{t+1}^{j}$. The relative motion model 〈i,j〉 can be used to predict the mis-detected target *j* according to the state of target *j* at previous time.

For both relative-motion models and the previous targets’ states, the motion transition is expressed by:

(17) $$\begin{array}{l} o_{t+1}^{i}=f(o_{t}^{j},\langle i,j\rangle )+w\\ \;=F{\left[[x_{t}^{j},y_{t}^{j},z_{t}^{j},{\dot{x}}_{t}^{j},{\dot{y}}_{t}^{j},{\dot{z}}_{t}^{j}]+r_{t}^{\langle i,j\rangle }\right]}^{T}+w \end{array}$$

where $F=\left[\begin{array}{cccccc} 1 & 0 & 0 & 1 & 0 & 0\\ 0 & 1 & 0 & 0 & 1 & 0\\ 0 & 0 & 1 & 0 & 0 & 1\\ 0 & 0 & 0 & 1 & 0 & 0\\ 0 & 0 & 0 & 0 & 1 & 0\\ 0 & 0 & 0 & 0 & 0 & 1 \end{array}\right]$ is the transition matrix based on a constant velocity of motion model. The computation of object at y coordinate has no practical use and ***w*** is the assumed white Gaussian noise.

According to the relative-motion model, the state of target *i* can be solved by using the previous state of target *j* and the relative-motion model between them, which is more accurate and adaptable than targeting predicted directly by linear motion model. When target *i* is missed in the detection stage, its location can be predicted through target *j* according to the motion model, which effectively reduces the false tracking caused by missed detection. The target’s prior motion is written as:

(18) $$P(O_{t}|O_{t-1})=P(o_{t}^{i}|o_{t-1}^{j},\langle i,j\rangle )$$

Three motion priori were used to account for the smooth transition of camera, targets, and geometric features through time. The target’s and geometric features’ prior motions also allowed them to appear, interact, and disappear, which made the model more robust and flexible.

## 4. Implementation

The unified Bayesian model for multiple object tracking and camera estimation, has been presented in last section. The solution of this model can be obtained typically by the maximum-a-posteriori method. Because the number of targets and features are variable in the tracking process, RJ-MCMC particle filtering was employed to find the MAP solution of the posterior distribution. The advantages of the RJ-MCMC method made it particularly suitable for adding and removing targets via random jump proposal moves in different dimensions.

As illustrated in Equation (1), the goal of tracking is defined as finding the states that maximize the posterior configuration $\chi_{t}=\arg\max\;P(\chi_{t}{|I}_{1,\ldots ,t})$. To obtain the posterior, RJ-MCMC samples, the posteriors from 1 to t to obtain a number of samples to approximate the posterior $P(\chi_{t}{|I}_{1,\ldots ,t})\approx {\left\{\chi_{t}^{\left(r\right)}\right\}}_{r=1}^{n}$, where *n* is the number of samples and $\chi_{t}^{\left(r\right)}$ is the $r^{th}$ sample. If the set of samples at previous time *t* − 1 is provided, then the posterior distribution can be expressed as:

(19) $$P(\chi_{t}{|I}_{1,\ldots ,t})\propto P(I_{t}|\chi_{t})\sum_{r}P(\chi_{t}|\chi_{t-1}^{\left(r\right)})$$

### 4.1. Proposal Distribution

As discussed in the previous section, the configuration variables defined in this paper are composed of three parts $\chi_{t}=\{Z_{t},G_{t},\Theta_{t}\}$. We needed to sample from those three components, but they would converge very slowly to steady-state distribution if we sampled from the whole configuration, due to their high dimensionality. To solve the problem, we sampled only one variable at a time. For example, we chose only one type of parameter to perturb to generate a new sample. Usually, the RJMCMC method follows the Metropolis-Hasting rule to generate samples, and then accepts or rejects samples to construct the Markov chain $\left\{\chi_{t}^{\left(0\right)},\chi_{t}^{\left(1\right)},\ldots ,\chi_{t}^{\left(n\right)}\right\}$.

Let $Q(\chi_{t}^{\prime },\chi_{t})$ be the proposal distribution; we used probability $q_{O}$ to perturb the target proposal $Q_{O}$, probability $q_{G}$ to perturb the geometric feature proposal $Q_{G}$, and probability $q_{\Theta }$ to perturb the camera proposal $Q_{\Theta }$. Then, the proposal distribution can be written as:

(20) $$Q(\chi_{t}^{\prime },\chi_{t})=q_{O}Q_{O}(\chi_{t}^{\prime },\chi_{t})+q_{G}Q_{G}(\chi_{t}^{\prime },\chi_{t})+q_{\Theta }Q_{\Theta }(\chi_{t}^{\prime },\chi_{t})$$

For example, with the probability $q_{O}$, the target proposal was randomly chosen; upon perturbation, the new configuration will be $\chi_{t}^{\left(r+1\right)}=\{O_{t}{}^{\left(r+1\right)},G_{t}{}^{\left(r\right)},\Theta_{t}{}^{\left(r\right)}\}$. Only a single target’s state has been changed in $O_{t}{}^{\left(r+1\right)}$, and the other terms will remain unchanged.

The three proposal distributions in Equation (20) are presented as follows.

(1) Target proposal distribution

Five jump moves for the target-state transition were defined: Stay, Leave, Add, Delete, and Update. Stay/Leave was defined as targets staying or leaving from the tracking scene with variable dimensionality, and Add/Delete was defined as new entering targets or false alarms in the tracking scene. Those two couple moves were designed to be reversible counterparts to each other; this guaranteed that the Markov Chain satisfied the detailed balance condition. The update move was defined for targets’ position updates. Overall, those five jump moves covered all conditions of target movement. During exploration, one of the five moves was randomly chosen with probabilities of $p_{s}$, $p_{l}$, $p_{a}$, $p_{d}$, $p_{u}$, respectively. In Table 1, the proposal distribution for each jump move and corresponding targets that suit for this jump move are listed.

$P(O_{t}{}^{i}|O_{t-1}^{j})$ will be set to 1 when there is no corresponding detection, or it will be set to $\frac{1}{2}\left[P(O_{t}{}^{i}|O_{t-1}^{j})+P(O_{t}{}^{i}|O_{t}^{i})\right]$, otherwise.

(2) Proposal Distribution of Geometric Feature

Because the geometric features state $G_{t}$ is also a high dimensional vector with variable dimensionality same as the target state, we used the RJ-MCMC method to sample from the geometric features to generate proposal distribution. However, only three jump moves (Stay, Leave, and Update) were necessary to update the geometric feature states, and with the same strategy as the target proposal distribution, one of the moves was randomly chosen with a probability of p*_s_*, p*_l_* and p*_u_*, respectively, to generate a proposal. Because of the validity of features needs to compare the previous features, we did not need the Add and Delete moves to add new features. All of the newly detected features were automatically added to the feature set in the current frame, and the validity of features in the subsequent frames was checked by comparing the observed and predicted positions with Stay and Leave moves. Table 2 lists each jump move’s proposal and the corresponding geometric features that suit this jump move.

(3) Camera State Proposal Distribution

Because the state dimensionality of the camera was kept constant, its state proposal distribution $Q(\Theta_{t}^{i(r+1)},\Theta_{t}^{i(r)})$ can be expressed by a simple normal distribution as:

(21) $$N(\Theta_{t}^{\left(r+1\right)};\Theta_{t}^{\left(r\right)};\sum_{\Theta })$$

### 4.2. Acceptance Ratio

In the traditional MH algorithm [43], for the acceptance ratio, only the ratio of the likelihood of the proposed configuration to the likelihood of the previous configuration was considered. We extended it to a product of three terms as follows:

(22) $$a=\frac{P(I_{t}|\chi_{t}^{\left(r+1\right)})}{P(I_{t}|\chi_{t}^{\left(r\right)})}\frac{P(\chi_{t}^{\left(r+1\right)}|I_{1,2,\ldots ,t})}{P(\chi_{t}^{\left(r\right)}|I_{1,2,\ldots ,t})}\frac{Q(\chi_{t}^{\left(r\right)};\chi_{t}^{\left(r+1\right)})}{Q(\chi_{t}^{\left(r+1\right)};\chi_{t}^{\left(r\right)})}$$

On the right hand side of Formula (22), the first term refers to the ratio of image likelihoods between the proposed configuration and the previous configuration, the second term describes the ratio of approximated predictions and the last term stands for the ratio of proposal distributions. By adding the ratio of prediction and proposal distribution, the results can be improved.

## 5. Experiments and Discussion

To evaluate the performance of the proposed tracking method, two kinds of dataset were used: the dataset collected by us in the campus, and some benchmark dataset with a moving camera, such as ETH-Bahnhof, ETH-Linthescher, ETH-Sunnyday [44], MOT16-05, and MOT16-11 [45]. For all experiments, we assumed that an initial camera configuration was provided, which included the focal length and height of the camera. Because the camera was not calibrated for the dataset collected by ourself, the camera configuration parameters were initialized with rough values. For the ETH dataset and MOT16s, the calibrated parameters were provided and used as the default value in the experiments. For the video sequences collected by ourselves, only the camera height and focal length needed to be roughly set. Accurate camera calibration was not necessary for the proposed method. The focal length can be set according to the datasheet of the camera, and the camera height is set to the value estimated by the eye in the experiments. The proposed methods would be applied in any flat environment.

Many evaluation metrics for quantitative evaluation of MOT have been proposed in the past. In this paper, we followed the evaluation metrics suggested in the MOT2D benchmark challenge [44,45].

These metrics could evaluate a method from different aspects, and their definitions are listed as follows:

The tracking accuracy can be evaluated by multiple object tracking accuracy (MOTA). It is widely used because of its expressiveness, as it includes three sources of errors, which are defined as:

$$MOTA=1-\frac{\sum_{t}\left(FN_{t}+FP_{t}+IDSW_{t}\right)}{\sum_{t}GT_{t}}$$

where *t* is the frame index, FP denotes false positives, which means the output is a false alarm, FN is false negatives; a target that is missed by any hypothesis is a false negative. GT is the number of ground truth objects. IDSW is the number of target identification (ID) switches. The MOTA score can give a good indication of the overall performance.

The tracking precision is evaluated by multiple object tracking precision (MOTP). It is the average dissimilarity between all true positives and their corresponding ground truth targets. It is computed as:

$$MOTP=\frac{\sum_{t,i}d_{t,i}}{\sum_{t}c_{t}}$$

where $c_{t}$ denotes the number of matches in frame *t* and $d_{t,i}$ is the bounding box overlap of target *i* related to the ground truth. MOTP measures the localization precision, and describes the average overlap between all correctly matched hypotheses and their respective objects ranges between 50% and 100%.

The tracking quality for each ground truth trajectory can be classified into mostly tracked (*MT*), partially tracked (*PT*), or mostly lost (*ML*). A target is mostly tracked, which defines as it is successfully tracked for at least 80% of its life span. If a target track is only recovered for less than 20% of its total length, then it is mostly lost (*ML*). All other tracks are defined as partially tracked. Usually, they are expressed as a ratio to the total number of ground truth trajectories.

Recall is defined as the ratio of correctly matched detections to the total detections in ground truth.

Precision is defined as the ratio of the correctly matched detections to total detections in the tracking result.

FAR is defined as the ratio of the false alarms per frame.

Currently, most of the MOT methods use a static camera; only few of them are moving-camera-based. Among them, continuous energy minimization (CEM) [30] and RJMCMC [27] are two typical methods with a moving camera; both the RMN method [22] and structural constraint event aggregation (SCEA) method [26] make use of the 2D motion context for the MOT. The CEM method belongs to a kind of batch-optimization method, and all of the historical data are used for the offline tracking optimization, which makes the data association robust, and the problem of target ID switch is improved. On the other hand, the RJMCMC method is an online optimization mode; the proposed method in this paper also employs the RJMCMC framework to find the maximum-a-posterior (MAP) solution. Thus, we made a thorough comparison among five methods: CEM, RJMCMC, RMN, SCEA and the proposed method. Because the proposed method was based on RJ-MCMC, we took it as the baseline method.

Table 2 shows the comparison results for the benchmark dataset, ETH-Bahnhof, ETH-Sunnyday, MOT16-05, and MOT16-11. The proposed method outperformed the other four methods in precision, FAR, MOTA, and FP with the ETH-Bahnhof dataset, especially in MOTA and precision, though the CEM method was globally optimized. This is because the proposed algorithm can pass the 3D motion model to track the target in the detection process, which can improve the tracking accuracy. Because only the previous frame information was considered for online purposes in the proposed method, there were more target identity switch errors than in the CEM method, but fewer than in the RJMCMC method. At the same time, it can be seen that the overall performances in the proposed method were better than in the RJMCMC method. It confirms that by exploiting the 3D relative motion model, the tracking performance was improved greatly.

For the experiment results with the ETH-Sunnyday, MOT16-05, and MOT16-11 dataset, the proposed method in MT, FN, and Recall was better than the CEM and RJMCMC algorithms. Because FN represents the number of missed target tracks, the results showed that the proposed method can greatly reduce missed detection by making use of the relative-motion model. Compared with the motion context methods (SCEA_ver1 and RMOT), the indexes of recall and MOTP for both methods were outperformed compared to the proposed method, because they exploited the motion context of all targets and we only exploited the nearest target’s motion context for calculation. There were some indexes of the proposed method that were higher than those two methods, and showed in bold in Table 3. But we also found that the proposed method might fail to predict target if the target is miss-detected for several frames. The feature work will aim at robust tracking methods account for unreliable detection.

**Figure 5:**
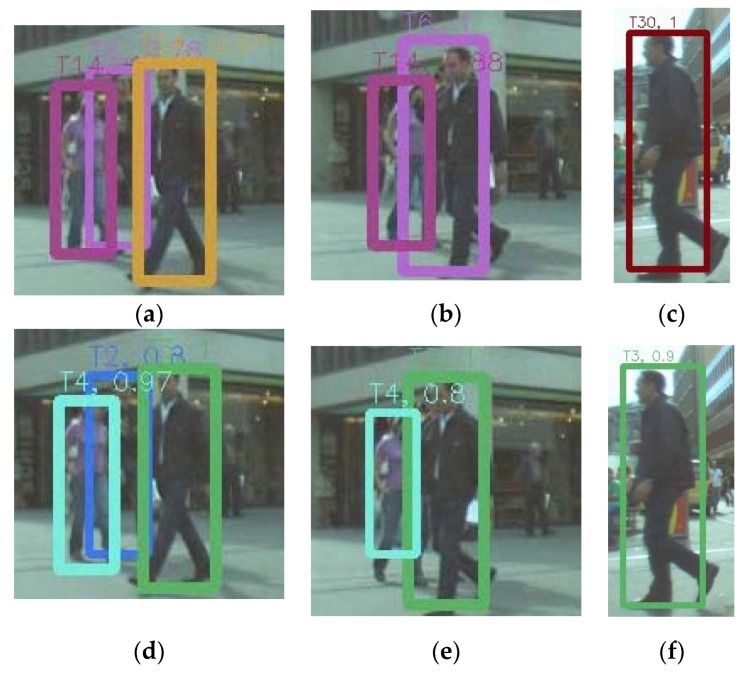
A qualitative comparison between the proposed method and the reversible jump Markov chain Monte Carlo (RJMCMC) algorithm with the ETH-Linthescher video. In this figure, (**a**–**c**) show the results obtained by the RJMCMC method in frames No. 32, 34, and 54; (**d**–**f**) respectively show the correspondent results with the proposed method.

Because the particle filter was exploited to solve the posterior estimation problem both in the proposed and RJ-MCMC method, we conducted some qualitative comparison experiments between the proposed and the RJMCMC method with the ETH-Linthescher sequence, and the same detection method was used. Figure 5a–c shows the tracking results of the 32th, 34th and 54th frame, respectively, in the ETH-Linthescher sequence. When occlusion occurred, the target ID switch happened in the RJMCMC method, but for the proposed method, targets ID did not switch, as shown in (e). Similarly, the target ID switched in the 54th frame in the RJMCMC method, but our method could keep the correct target ID. It shows that the proposed method prevented the tracking drift when occlusion occurred, because it benefitted from the proposed relative motion model.

Generally, a weakness of tracking by detection is that the tracking result is heavily dependent on the reliability of the detection module. If the detection module fails to detect the target, then the target may not be tracked, and this leads to the generation of track fragments or tracking drift. To alleviate this issue, the proposed method exploits the relative-motion model in 3D space to describe the motion constraints among targets, which can improve the performance. Some demonstrative experiments were conducted with ETH-Sunnyday, ETH-Bahnhof, and the datasets we collected.

**Figure 6:**
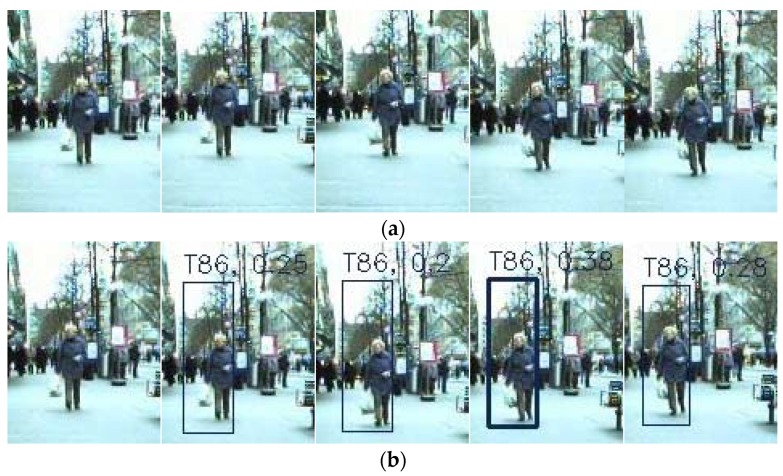
Comparison of the tracking results between RJMCMC and the proposed method with ETH-Bahnhof. (**a**) Detection results and tracking results of the RJMCMC method; (**b**) Detection and tracking results of the proposed method.

As shown in Figure 6, both the RJMCMC method and the proposed method failed to detect the old woman, this led to the fragments showed in Figure 6a because the RJMCMC method could not track the woman. However, the proposed method could work successfully, and avoid generating the tracking fragments when mis-detection occurred.

**Figure 7:**
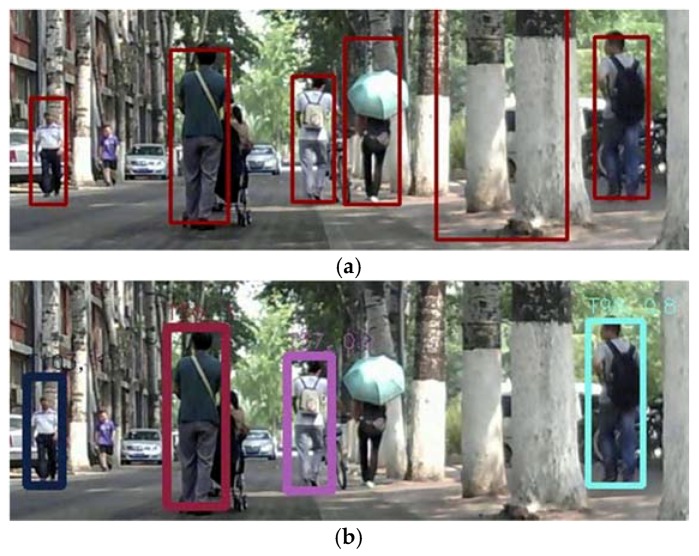

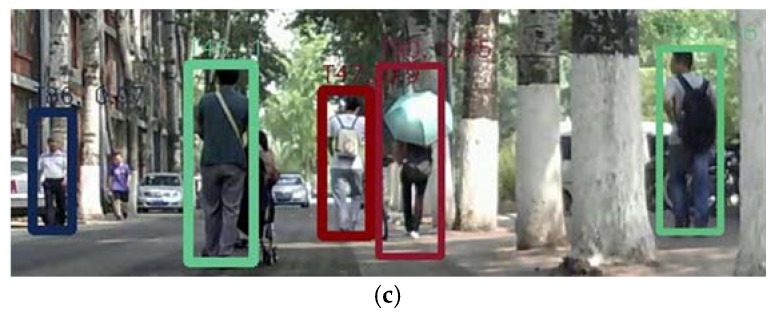
Comparison between RJMCMC and the proposed method with the video collected in our campus. (**a**) The detection results. A wrong detection result exists indicated by the largest bounding box. (**b**) The results of the RJMCMC method; the woman with umbrella is lost. (**c**) The good results of the proposed method.

Beside the experiments on these standard datasets, we also conducted some experiments with the video data collected in our campus. As shown in Figure 7, it was challenging for the tracking because of the illumination change, scale variation of the target, and the appearance differences. Because there was no ground truth data available, only some qualitative results could be provided. In this figure, given the same detection result, the tracking result of the proposed algorithm outperformed the RJMCMC method. Figure 7a shows the detection results, which include a wrong detection result (indicated by the largest bounding box). Both the proposed and RJMCMC method could remove it in the tracking process. However, as shown in Figure 7b, the RJMCMC method fail to track the woman with an umbrella. The proposed method worked well in this case.

**Figure 8:**
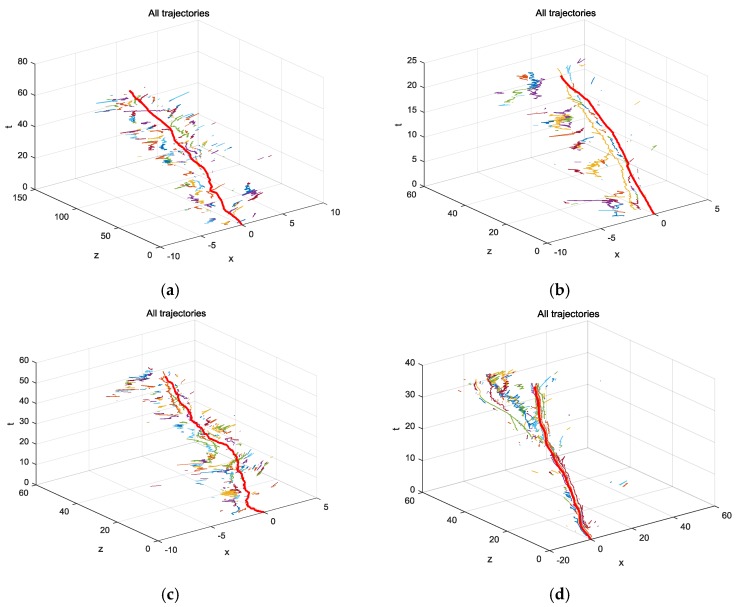
The camera trajectory (long red lines) and targets’ trajectories (short multi-color lines) of four video sequences (**a**) ETH-Bahnhof. (**b**) ETH-Sunnyday. (**c**) MOT16-05. (**d**) MOT16-11.

Figure 8 shows the results of the camera and target trajectories in terms of time with four video sequences, ETH-Bahnhof, ETH-Sunnyday, MOT16-05, MOT16-11. In these figures, the plane (*x*, *z*) was defined according to the camera coordinate in the very first frame of each sequence. It can be seen that although no ground truth is available for the camera motion, the camera motion estimated here qualitatively matched what we saw in the video.

All the experiments are run on an Intel core i5 personal computer (PC) with 8 GB memory, and Visual Studio 2012 and Opencv2.4.9 were used, the sample particles were set to 5000. Currently, the average computation time of detection and tracking was approximately 0.17 s without any code optimization. Most of the time was spent by the detector, about 91% in total. It is mainly because the DPM detector is time-consuming.

The experiments also showed that, because the proposed method is based on the principle of tracking-by-detection, it could not track the target mis-detected when it first appeared. As showed in Figure 7a,c, the person in the purple T-shirt was not detected at first, then he could not be tracked consequently. A more powerful detector may improve this case.

## 6. Conclusions

In this paper, a Bayes posterior estimation-based MOT method is presented under the framework of tracking by detection. We exploit the 3D motion context information from multiple objects, which describes the relative movements among the targets, to improve the tracklets and tracking drifting caused by occlusion and mis-detections. Also, we formulate the target tracking and camera motion estimation as a sequential Bayesian framework which is solved by RJMCMC particle filtering; the camera motion estimation provides the opportunity for 3D relative-movement estimation among targets. The detailed models for the posterior estimation are introduced. Some quantitative experiments with five benchmark datasets are conducted. Qualitative experiments with the benchmark dataset and the campus video we collected are also carried out. All of the experiments confirm that the proposed method is outperformed in handling the track tracklets due to occlusion and unreliable detections.

The proposed method employs the tracking-by-detection framework, so it heavily depends on the detector. Additionally, because the DPM feature are used in the proposed detector, this increases the computing complexity. The next works include efficiency optimization and application of some CNN-based feature detection in the proposed framework.

## Figures and Tables

**Table 1:** The proposal of targets’ jump moves.

Jump Moves	Corresponding Targets	Proposal
Stay: $S_{t}^{\left(r\right)}$	$S_{t}^{\left(r\right)}=O_{t-1}\backslash O_{t}^{\left(r\right)}$	$Q_{S}(O_{t}^{\left(r+1\right)},O_{t}^{\left(r\right)})=\left\{ \begin{array}{cc} \frac{1}{\left|S_{t}^{\left(r\right)}\right|}Q_{i}{\left(O_{t}^{i(r+1)}<i,j>\right)}^{1} & \mathrm{if}\;i\;\mathrm{in}\;S_{t}^{\left(r\right)}\\ 0 & \mathrm{otherwise} \end{array}\right.$
Leave: $L_{t}^{\left(r\right)}$	$L_{t}^{\left(r\right)}=O_{t}^{\left(r\right)}\cap O_{t-1}$	$Q_{L}(O_{t}^{\left(r+1\right)},O_{t}^{\left(r\right)})=\left\{ \begin{array}{cc} \frac{1}{\left|L_{t}^{\left(r\right)}\right|} & \mathrm{if}\;i\;\mathrm{in}\;L_{t}^{\left(r\right)}\\ 0 & \mathrm{otherwise} \end{array}\right.$
Add: $A_{t}^{\left(r\right)}$	$A_{t}^{\left(r\right)}=X_{t}^{new}\backslash O_{t}^{\left(r\right)}$	$Q_{A}(O_{t}^{\left(r+1\right)},O_{t}^{\left(r\right)})=\left\{ \begin{array}{cc} \frac{1}{\left|A_{t}^{\left(r\right)}\right|}P(O_{t}^{i}|X_{t}^{i}) & \mathrm{if}\;i\;\mathrm{in}\;A_{t}^{\left(r\right)}\\ 0 & \mathrm{otherwise} \end{array}\right.$
Delete: $D_{t}^{\left(r\right)}$	$D_{t}^{\left(r\right)}=X_{t}^{new}\cap O_{t}^{\left(r\right)}$	$Q_{D}(O_{t}^{\left(r+1\right)},O_{t}^{\left(r\right)})=\left\{ \begin{array}{cc} \frac{1}{\left|D_{t}^{\left(r\right)}\right|} & \mathrm{if}\;i\;\mathrm{in}\;D_{t}^{\left(r\right)}\\ 0 & \mathrm{otherwise} \end{array}\right.$
Update: $K_{t}^{\left(r\right)}$	$K_{t}^{\left(r\right)}=L_{t}^{\left(r\right)}\cup D_{t}^{\left(r\right)}$	$Q_{U}(O_{t}^{\left(r+1\right)},O_{t}^{\left(r\right)})=\left\{ \begin{array}{cc} \frac{1}{\left|U_{t}^{\left(r\right)}\right|}Q(O_{t}^{i(r+1)},O_{t}^{i(r)}) & \mathrm{if}\;i\;\mathrm{in}\;K_{t}^{\left(r\right)}\\ 0 & \mathrm{otherwise} \end{array}\right.$

**Table 2:** The proposal of geometric features’ jump moves.

Jump Moves	Corresponding Features	Proposal
Stay: $S_{t}^{\left(r\right)}$	$S_{t}^{\left(r\right)}=G_{t-1}\backslash G_{t}^{\left(r\right)}$	$Q_{S}(G_{t}^{\left(r+1\right)},G_{t}^{\left(r\right)})=\left\{ \begin{array}{cc} \frac{1}{\left|S_{t}^{\left(r\right)}\right|}P_{cons}(G_{t}^{j(r+1)},G_{t}^{j(r)}) & \mathrm{if}\;i\;\mathrm{in}\;S_{t}^{\left(r\right)}\\ 0 & \mathrm{otherwise} \end{array}\right.$
Leave: $L_{t}^{\left(r\right)}$	$L_{t}^{\left(r\right)}=G_{t}^{\left(r\right)}\cap G_{t-1}$	$Q_{L}(G_{t}^{\left(r+1\right)},G_{t}^{\left(r\right)})=\left\{ \begin{array}{cc} \frac{1}{\left|L_{t}^{\left(r\right)}\right|} & \mathrm{if}\;i\;\mathrm{in}\;L_{t}^{\left(r\right)}\\ 0 & \mathrm{otherwise} \end{array}\right.$
Update: $E_{t}^{\left(r\right)}$	$E_{t}^{}=G_{t}^{\left(r\right)}\backslash G_{t-1}$	$Q_{U}(Z_{t}^{\left(r+1\right)},Z_{t}^{\left(r\right)})=\left\{ \begin{array}{cc} \frac{1}{\left|E_{t}^{\left(r\right)}\right|}Q(G_{t}^{i(r+1)},G_{t}^{i(r)}) & \mathrm{if}\;i\;\mathrm{in}\;E_{t}^{}\\ 0 & \mathrm{otherwise} \end{array}\right.$

**Table 3:** Performance comparison of tracking in different video sequences.

Dataset	Method	Recall/%	Precision/%	FAR	MT%	PT%	ML%	FP	FN	IDs	MOTA/%	MOTP/%
ETH-	CEM	58.9	72	1.24	23.39	32.74	43.86	1241	2223	16	35.7	71.8
Bahnhof	RJMCMC	51.7	81	0.66	19.29	29.82	50.88	657	2614	102	37.7	66.7
	SCEA_ver1	73.2	64.2	1.11	19.30	13.45	14.62	1110	727	7	32.1	75.7
	RMOT	79.7	54.5	3.6	58.48	22.22	19.30	3599	1101	17	12.9	73
	Ours	55	82.9	0.61	19.88	33.33	46.78	615	2435	89	42	67.9
ETH-	CEM	37.1	86.4	0.31	16.67	20	63.33	108	1169	1	31.2	76
Sunnyday	RJMCMC	67	86.8	0.53	16.67	66.67	16.67	189	613	43	54.5	67.8
	SCEA_ver1	72.3	87.6	0.54	19	26.67	10	190	514	11	61.5	77.4
	RMOT	86.7	81.4	1.04	21	23.33	6.67	367	248	8	66.5	74.2
	Ours	69.2	85	0.64	23.33	63.33	13.33	226	573	61	53.7	68
MOT16-05	CEM	32.3	87.8	0.36	3.20	40.80%	56%	305	4616	65	26.9	74.5
	RJMCMC	34.4	77.2	0.83	5.60	46.40%	48%	694	4471	127	22.4	69.4
	SCEA_ver1	40.7	89.5	0.39	10	58%	57%	327	4045	34	35.4	76.1
	RMOT	44.2	57.4	2.68	8	68%	49%	2240	3802	135	9.4	71.1
	Ours	36.1	82	0.65	5.60	50.40%	44%	540	4359	143	26	69.5
MOT16-11	CEM	43.2	86.9	0.66	7.24	34.78%	57.97%	595	5212	27	36.4	78.4
	RJMCMC	46.9	87	0.72	5.80	46.38%	47.82	644	4867	144	38.4	73.6
	SCEA_ver1	28.9	88.2	0.39	8.96%	23.88%	70.15%	354	6520	8	25	79.5
	RMOT	44.2	57.4	5.19	16.42%	44.78%	43.28%	4674	3828	112	6.1	73.8
	Ours	49.4	85.1	0.88	7.24%	46.38%	46.38%	794	4645	168	38.9	72.3

## References

[1] Pirsiavash H., Ramanan D., Fowlkes C.C. Globally-optimal greedy algorithms for tracking a variable number of objects. Proceedings of the IEEE Conference on Computer Vision and Pattern Recognition.

[2] Dehghan A., Modiri Assari S., Shah M. GMMCP tracker: Globally optimal Generalized Maximum Multi Clique problem for multiple object tracking. Proceedings of the IEEE International Conference on Computer Vision and Pattern Recognition.

[3] Milan A., Lealtaixe L., Schindler K. Joint tracking and segmentation of multiple targets. Proceedings of the IEEE Conference on Computer Vision and Pattern Recognition.

[4] Le N., Heili A., Odobez M. Long-term time-sensitive costs for CRF-based tracking by detection. Proceedings of the ECCV 2016 Workshops.

[5] Milan A., Schindler K., Roth S. (2016). Multi-target tracking by discrete-continuous energy minimization. Pattern Anal. Mach. Intell..

[6] Tang S., Andres B., Andriluka M., Schiele B. Multi-person tracking by multicuts and deep matching. Proceedings of the ECCV 2016 Workshops.

[7] Leal-Taixé L., Canton-Ferrer C., Schindler K. Learning by tracking: Siamese CNN for robust target association. Proceedings of the IEEE Computer Society Conference on Computer Vision and Pattern Recognition Workshops.

[8] Yu Q., Medioni G., Cohen I. Multiple Target Tracking Using Spatio-Temporal Markov Chain Monte Carlo Data Association. Proceedings of the Computer Vision and Pattern Recognition.

[9] Song B., Jeng T.Y., Staudt E. A Stochastic Graph Evolution Framework for Robust Multi-target Tracking. Proceedings of the European Conference on Computer Vision.

[10] Li X., Wang K., Wang W. A multiple object tracking method using Kalman filter. Proceedings of the IEEE International Conference on Information and Automation.

[11] Azari M., Seyfi A., Rezaie A.H. Real Time Multiple Object Tracking and Occlusion Reasoning Using Adaptive Kalman Filters. Proceedings of the 2011 7th Iranian Conference on Machine Vision and Image Processing.

[12] Zhang H., Yang J., Ge H. An improved GM-PHD tracker with track management for multiple target tracking. Proceedings of the International Conference on Control, Automation and Information Sciences.

[13] Khazaei M., Jamzad M. Multiple human tracking using PHD filter in distributed camera network. Proceedings of the International Conference on Computer and Knowledge Engineering.

[14] Bae S.H., Yoon K.J. (2017). Confidence-Based Data Association and Discriminative Deep Appearance Learning for Robust Online Multi-Object Tracking. IEEE Trans. Pattern Anal. Mach. Intell..

[15] Kieritz H., Becker S., Hübner W., Arens M. Online multi-person tracking using integral channel features. Proceedings of the IEEE International Conference on Advanced Video- and Signal-Based Surveillance.

[16] Yang M., Jia Y. (2016). Temporal Dynamic Appearance Modeling for Online Multi-Person Tracking. Comp. Vis. Image Underst..

[17] Sadeghian A., Alahi A., Savarese S. Tracking the Untrackable: Learning To Track Multiple Cues with Long-Term Dependencies. Proceedings of the IEEE International Conference on Computer Vision.

[18] Kristan M., Matas J., Leonardis A., Vojíř T., Pflugfelder R., Fernandez G., Nebehay G., Porikli F., Čehovin L. (2016). A Novel Performance Evaluation Methodology for Single-Target Trackers. IEEE Trans. Pattern Anal. Mach. Intell..

[19] Zhang B., Li Z., Cao X., Ye Q., Chen C., Shen L., Perina A., Ji R. (2017). Output constraint transfer for kernelized correlation filter in tracking. IEEE Trans. Syst. Man Cybern. Syst..

[20] Zhang B., Luan S., Chen C., Han J., Wang W., Perina A., Shao L. (2017). Latent constrained correlation filter. IEEE Trans. Image Proc..

[21] Ma C., Huang J.B., Yang X., Yang M.H. (2018). Robust visual tracking via hierarchical convolutional features. IEEE Trans. Pattern Anal. Mach. Intell..

[22] Ju H.Y., Yang M.H., Lim J. Bayesian Multi-object Tracking Using Motion Context from Multiple Objects. Proceedings of the IEEE Winter Conference on Applications of Computer Vision.

[23] Breitenstein M.D., Reichlin F., Leibe B. (2011). Online Multi-person Tracking-by-Detection from a Single, Uncalibrated Camera. IEEE Trans. Pattern Anal. Mach. Intell..

[24] Sanchez-Matilla R., Poiesi F., Cavallaro A. Online Multi-target Tracking with Strong and Weak Detections. Proceedings of the European Conference on Computer Vision.

[25] Leal-Taixé L., Fenzi M., Kuznetsova A., Rosenhahn B., Savarese S. Learning an image-based motion context for multiple people tracking. Proceedings of the IEEE Conference on Computer Vision and Pattern Recognition.

[26] Ju H.Y., Lee C.R., Yang M.H. Online Multi-object Tracking via Structural Constraint Event Aggregation. Proceedings of the Computer Vision and Pattern Recognition.

[27] Choi W., Pantofaru C., Savarese S. (2013). A General Framework for Tracking Multiple People from a Moving Camera. IEEE Trans. Pattern Anal. Mach. Intell..

[28] Romero-Cano V., Agamennoni G., Nieto J. A variational approach to simultaneous tracking and classification of multiple objects. Proceedings of the International Conference on Information Fusion (FUSION).

[29] Romero-Cano V., Agamennoni G., Nieto J. (2015). A variational approach to simultaneous multi-object tracking and classification. Int. J. Robot. Res..

[30] Milan A., Roth S., Schindler K. (2014). Continuous energy minimization for multi-target tracking. IEEE Trans. Pattern Anal. Mach. Intell..

[31] Yang B., Huang C., Nevatia R. Learning affinities and dependencies for multi-target tracking using a CRF model. Proceedings of the IEEE Conference on Computer Vision and Pattern Recognition.

[32] Mclaughlin N., Martinez Del Rincon J., Miller P. Enhancing Linear Programming with Motion Modeling for Multi-target Tracking. Proceedings of the Winter Conference on Applications of Computer Vision.

[33] Yang B., Nevatia R. Multi-target tracking by online learning of non-linear motion patterns and robust appearance models. Proceedings of the IEEE Conference on Computer Vision and Pattern Recognition.

[34] Dicle C., Sznaier M., Camps O. The way they move: Tracking multiple targets with similar appearance. Proceedings of the IEEE International Conference on Computer Vision.

[35] Yang B., Nevatia R. (2014). Multi-Target Tracking by Online Learning a CRF Model of Appearance and Motion Patterns. Int. J. Comp. Vis..

[36] Choi W., Savarese S. Multiple target tracking in world coordinate with single, minimally calibrated camera. Proceedings of the European Conference on Computer Vision.

[37] Bay H., Ess A., Tuytelaars T. (2008). Speeded-Up Robust Features. Comp. Vis. Image Underst..

[38] Tomasi C. (1991). Detection and tracking of point features. Tech. Rep..

[39] Hoiem D., Efros A.A., Hebert M. Putting Objects in Perspective. Proceedings of the 2006 IEEE Computer Society Conference on Computer Vision and Pattern Recognition (CVPR’06).

[40] Wu B., Nevatia R. (2007). Detection and tracking of multiple, partially occluded humans by Bayesian combination of edgelet based part detectors. Int. J. Comp. Vis..

[41] Felzenszwalb P.F., Girshick R.B., McAllester D., Ramanan D. (2010). Object detection with discriminatively trained part-based models. IEEE Trans. Pattern Anal. Mach. Intell..

[42] Comaniciu D., Meer P. (2002). Mean Shift: A robust approach toward feature space analysis. IEEE Trans. Pattern Anal. Mach. Intell..

[43] Robert C.P., Casella G. (2000). Monte Carlo Statistical Method. Technometrics.

[44] Lealtaixé L., Milan A., Reid I. (2015). MOT Challenge 2015: Towards a Benchmark for Multi-Target Tracking. arXiv.

[45] Milan A., Leal-Taixé L., Reid I., Roth S., Schindler K. (2016). MOT16: A Benchmark for Multi-Object Tracking. arXiv.

